# Comparison of echocardiographic and invasive measures of volaemia and cardiac performance in critically ill patients

**DOI:** 10.1038/s41598-020-61761-1

**Published:** 2020-03-17

**Authors:** Konstantin Yastrebov, Anders Aneman, Luis Schulz, Thomas Hamp, Peter McCanny, Geoffrey Parkin, John Myburgh

**Affiliations:** 1 Department of Intensive Care, The St George Hospital, Sydney, Australia; 2 The University of New South Wales, Sydney, Australia; 3 Intensive Care Unit, Liverpool Hospital, Sydney, Australia; 4 Department of Anaesthesia, Intensive Care Medicine and Pain Medicine, Medical University of Vienna, Vienna, Austria; 5 Intensive Care Unit, Monash Medical Centre, Melbourne, Australia; 6 Monash University, Melbourne, Australia; 7 Critical Care Division, The George Institute for Global Health, Sydney, Australia

**Keywords:** Cardiology, Circulation

## Abstract

Echocardiographic measurements are used in critical care to evaluate volume status and cardiac performance. Mean systemic filling pressure and global heart efficiency measures intravascular volume and global heart function. This prospective study conducted in fifty haemodynamically stabilized, mechanically ventilated patients investigated relationships between static echocardiographic variables and estimates of global heart efficiency and mean systemic filling pressure. Results of univariate analysis demonstrated weak correlations between left ventricular end-diastolic volume index (r = 0.27, p = 0.04), right atrial volume index (rho = 0.31, p = 0.03) and analogue mean systemic filling pressure; moderate correlations between left ventricular ejection fraction (r = 0.31, p = 0.03), left ventricular global longitudinal strain (r = 0.36, p = 0.04), tricuspid annular plane systolic excursion (rho = 0.37, p = 0.01) and global heart efficiency. No significant correlations were demonstrated by multiple regression. Mean systemic filling pressure calculated with cardiac output measured by echocardiography demonstrated good agreement and correlation with invasive techniques (bias 0.52 ± 1.7 mmHg, limits of agreement −2.9 to 3.9 mmHg, r = 0.9, p < 0.001). Static echocardiographic variables did not reliably reflect the volume state as defined by estimates of mean systemic filling pressure. The agreement between static echocardiographic variables of cardiac performance and global heart efficiency lacked robustness. Echocardiographic measurements of cardiac output can be reliably used in calculation of mean systemic filling pressure.

## Introduction

In the clinical context, estimation of volume state and contractility derived by echocardiography are increasingly being advocated^1–3^, although these estimates have not been evaluated or compared to estimates of mean systemic filling pressure in the clinical setting.

The administration of intravenous fluid is one of the commonest interventions in acute medicine. There are wide variations in the estimation of volume state in clinical practice^4^. Given the adverse effects associated with the injudicious use of intravenous fluids^5^, there is an imperative to identify clinically applicable variables to accurately assess the volume state to facilitate the delivery of optimal clinical management.

The mean systemic filling pressure is a physiological variable that reflects the balance between the “stressed” intravascular volume and systemic cardiovascular compliance^6,7^. The routine measurement of mean systemic filling pressure is entering clinical practice but remains elusive while clinically applicable gold standard does not exist^7,8^. Measuring the equilibration pressure in an isolated upper limb^8^ and calculating an analogue pressure from a specific algorithm^9^ have been used to provide an indirect estimate of mean systemic filling pressure^10^. Despite ongoing debate regarding the role of mean systemic filling pressure in maintaining cardiac output^11^, recent research confirmed physiological concept of venous return driving pressure as the difference between mean systemic filling pressure and right atrial pressure, further emphasising that analogue estimate could accurately track dynamic changes of zero-flow measurements of mean systemic filling pressure, while inspiratory flow manoeuvres produced clinically unacceptable bias^12^. Analogue mean systemic filling pressure was therefore used as a “pragmatic” clinical reference standard.

The global heart efficiency (Eh) may be calculated by the difference between mean systemic filling and right atrial pressure divided by mean systemic filling pressure^9^. Eh is dimensionless. The monitoring system incorporating equations for continuous calculation of the analogue mean systemic filling pressure and heart efficiency used in this investigation has been previously investigated in clinical setting with promising results^13,14^. However, this concept remains unfamiliar to the wide audience, while pathophysiological meaning of global heart efficiency and its relationship with conventional variables of cardiac performance require further investigation. The concepts of mean systemic filling pressure and global heart efficiency may offer alternatives to the contemporary goals for haemodynamic management.

We hypothesised that there was a definable relationship between echocardiographically derived variables of volume state and estimates of mean systemic filling pressure and between cardiac systolic function and estimates of global heart efficiency.

## Results

A total of 50 patients were enrolled between February 2016 and November 2017. The CONSORT diagram is presented in Figure E1 in the Online Data Supplement. Patient characteristics are presented in Table 1. Haemodynamic variables are presented in Table 2. The complete set of echocardiographic variables are presented in the Table E1 in the Online Data Supplement.s

**Table 1:** Patients characteristics (N = 50 patients).

VARIABLE	Values
Age (years)	68 (8.8)
Admission type, n (%)	
Medical	8 (16%)
Sepsis	2 (4%)
Surgical	42 (84%)
Cardiothoracic surgery	39 (78%)
APACHE II	18 [15–23]
Weight (kg)	82 (18)
Height (cm)	168 [163–177]
Body Mass Index (kg/m^2^)	28 [26–32]
Body Surface Area (m^2^)	1.9 (0.25)
Cardiovascular medications, n (%)	
Adrenaline	2/37 (5.4%)
Adrenaline + glyceryl trinitrate	1/37 (2.7%)
Adrenaline + levosimendan	1/37 (2.7%)
Dobutamine	4/37 (11%)
Glyceryl trinitrate	5/37 (14%)
Levosimendan	1/37 (2.7%)
Nitroprusside	1/37 (2.7%)
Noradrenaline	13/37 (35%)
Noradrenaline + Adrenaline	2/37 (5.4%)
Noradrenaline + dopamine	1/37 (2.7%)
Noradrenaline + vasopressin	2/37 (5.4%)
Noradrenaline + glyceryl trinitrate	1/37 (2.7%)
Noradrenaline + milrinone	2/37 (5.4%)
Noradrenaline + verapamil	1/37 (2.7%)

Values are mean (standard deviation) or median [interquartile range] unless indicated otherwise.

**Table 2:** Haemodynamic characteristics (N = 50 patients).

VARIABLE	Value
Heart rate (bpm)	77 (14)
Systolic blood pressure (mm Hg)	116 [104–127]
Diastolic blood pressure (mm Hg)	52 [47–57]
Mean arterial blood pressure (mm Hg)	72 [66–76]
Central venous pressure (mm Hg)	12 (3.9)
Cardiac index by thermodilution (L/min/m^2^)	2.7 (0.8)
Cardiac output by echocardiography (L/min/m^2^)	2.5 (0.8)
P_arm_ (mm Hg)	26 (5.2)
P_msa_ (cardiac output by thermodilution) (mm Hg)	19 (3.9)
P_msa_ (cardiac output by echocardiography) (mm Hg)	19 (3.7)

Values are mean (standard deviation) or median [interquartile range].

Definition of abbreviations: P_arm_, mean systemic filling pressure measured by the arm occlusion method; P_msa_, analogue mean systemic filling pressure.

### Volume measurements

**Figure 1:**
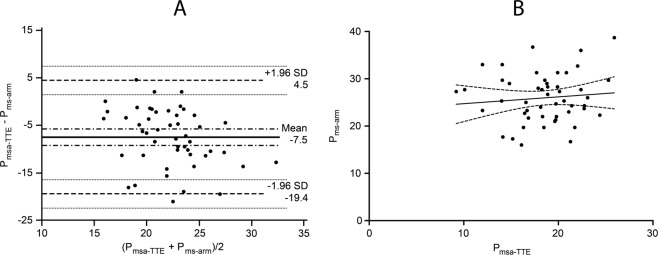
Graphic presentation of agreement and correlation between analogue mean systemic filling pressure calculated using echocardiography (P_msa-TTE_) and mean systemic filling pressure estimated by the upper limb stop flow technique (P_ms-arm_). Panel A: Bland Altman plot demonstrated a bias of −7.46 ± 6.1 mmHg (solid line) and the lower and upper limits of agreement at −19 to 4.5 mmHg (dashed lines). Panel B: Linear regression scatterplot graph. The correlation was r = 0.11, 95% CI −0.18 to 0.37, p = 0.48.

The mean systemic filling pressure estimated using echocardiography measurements of cardiac output was 18.5 ± 3.7 mmHg. Bland Altman analysis of mean systemic filling pressure estimated by the upper limb stop-flow technique and calculated analogue mean systemic filling pressure using transthoracic echocardiography measurements of cardiac output demonstrated a mean difference of −7.46 ± 6.1 mmHg with lower and upper limits of agreement between −19 to 4.5 mmHg. The correlation was r = 0.11, 95%CI −0.18 to 0.37, p = 0.48. (Fig. 1).

**Figure 2:**
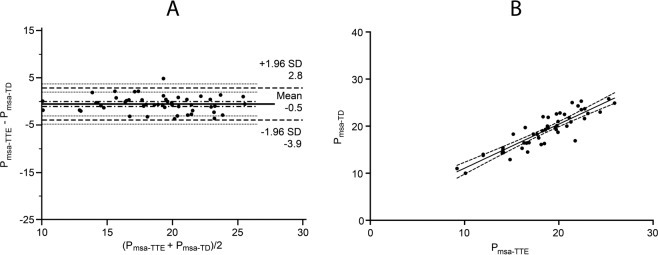
Agreement and correlation between analogue mean systemic filling pressure calculated using thermodilution-measured cardiac output (P_msa-TD_,) and analogue mean systemic filling pressure calculated using echocardiography-measured cardiac output (P_msa-TTE_). Panel A: Bland-Altman plot demonstrated a bias of 0.52 ± 1.7 mmHg and the lower and upper limits of agreement at −2.9 to 3.9 mmHg. Panel B: Linear regression scatterplot graph. The correlation was r = 0.90, 95% CI 0.82 to 0.94, p = <0.001.

Bland-Altman analysis of mean systemic filling pressure calculated based on cardiac output measurements using thermodilution methods and echocardiography demonstrated a mean difference of 0.52 ± 1.7 mmHg with lower and upper limits of agreement between −2.9 to 3.9 mmHg. The correlation was r = 0.90, 95% CI 0.82 to 0.94, p = <0.001 (Fig. 2).

Bland-Altman analysis of pressure gradient for venous return using the analogue mean systemic filling pressure calculated based on cardiac output measurements using thermodilution methods and echocardiography demonstrated a mean difference of 0.54 ± 1.5 mmHg with lower and upper limits of agreement between −2.3 and 3.4 mmHg. The correlation was r = 0.50, 95% CI 0.25 to 0.68, p = 0.0001.

The univariate analyses comparing mean systemic filling pressure, using isolated limb stop-flow technique and the analogue pressure calculated based on cardiac output measured by thermodilution and echocardiography, and the echocardiographic variables of volume status are reported in Table 3. Weak to moderate correlations of absolute and indexed right atrial volume were observed with mean systemic filling pressure estimated by all three techniques. Absolute and indexed left ventricular end-diastolic volume weakly correlated with mean systemic filling pressure calculated using cardiac output measurements by thermodilution and echocardiography.

**Table 3:** Univariate analysis of mean systemic filling pressure and echocardiographic variables used for assessments of intravascular/intracardiac filling status.

VARIABLE	P_ms_ estimated by the upper limb stop-flow technique	P_ms_ calculated using thermodilution measurements of CO	P_ms_ calculated using echocardiographic measurement of CO
LV end-diastolic volume index (ml/m^2^)	rho = 0.05 (p = 0.74)	**r** = **0.27** **(p = 0.04)**	**0.28** **(p** = **0.04)**
LV end-diastolic area (cm^2^)	rho = 0.06 (p = 0.68)	rho = 0.17 (p = 0.27)	rho = 0.15 (p = 0.34)
LV end-systolic volume index (ml/m^2^)	rho = 0.01 (p = 0.93)	r = 0.12 (p = 0.42)	r = 0.23 (p = 0.13)
LV end-systolic area (cm^2^)	rho = 0.12 (p = 0.44)	rho = 0.16 (p = 0.29)	rho = 0.14 (p = 0.37)
LA volume (ml/m^2^)	rho = 0.16 (p = 0.26)	rho = 0.13 (p = 0.26)	rho = 0.12 (p = 0.32)
RA volume (ml/m^2^)	**rho** = **0.33** **(p** = **0.02)**	**rho** = **0.31** **(p** = **0.03)**	**rho** = **0.29** **(p** = **0.04)**
IVC diameter (inspiration) (mm)	r = 0.03 (p = 0.87)	r = 0.22 (p = 0.15)	r = 0.24 (p = 0.12)
IVC diameter (expiration) (mm)	r = 0.09 (p = 0.56)	r = 0.16 (p = 0.30)	r = 0.23 (p = 0.13)
IVC distensibility index (%)	0.18 (p = 0.25)	0.24 (p = 0.12)	0.15 (p = 0.35)
E/e’	r = 0.05 (p = 0.76)	r = 0.08 (p = 0.58)	r = 0.03 (p = 0.93)

Correlations are described by Pearson (r) and Spearman (rho) with the p-values within brackets. Statistically significant results are depicted in bold.

Definition of abbreviations: LV = left ventricle; LA = left atrium; RA = right atrium; IVC = inferior vena cava; RV = right ventricle; E/e’ = early mitral diastolic inflow velocity to early diastolic mitral annular motion velocity ratio; CO = cardiac output.

Multivariate analysis of selected univariates did not demonstrate significant correlations between mean systemic filling pressure and echocardiographic variables (see Table E4 in the Online Data Supplement).

The results of mean systemic filling pressure measurements by three different techniques are summarised in Table E2 in the Online Data Supplement.

The mean ± SD systemic filling pressure estimated by the upper limb stop-flow technique was 26 ± 5.2 mmHg compared to the calculated analogue mean systemic filling pressure using thermodilution measurements of cardiac output of 19 ± 3.9 mmHg. The Bland Altman analysis demonstrated a mean difference of −6.9 ± 0.84 mmHg with lower and upper limits of agreement between −18 to 4.6 mm Hg. The correlation was r = 0.19, 95% CI −0.1 to 0.44, p = 0.20.

Agreements and correlations between estimates of mean systemic filling pressure by three different techniques are presented in Table E3 in the Online Data Supplement.

### Cardiac output

Bland-Altman analysis of cardiac index measured by thermodilution and transthoracic echocardiography demonstrated a mean difference of 0.26 ± 0.8 L/min/m^2^ with the lower and upper limits of agreement between −1.4 to 1.9 L/min/m^2^. The correlation was r = 0.47, 95%CI 0.23 to 0.67, p = 0.001.

### Global heart efficiency

The results of global heart efficiency calculations by three different techniques are summarised in Table E2 in the Online Data Supplement.

The global heart efficiency estimated by the upper limb stop flow technique was 0.51 ± 0.17 compared to the calculated using thermodilution measurements of cardiac output of 0.36 ± 0.12. Bland Altman analysis demonstrated a mean difference of −0.15 ± 0.12 with the lower and upper limits of agreement between −0.39 and 0.09. The correlation was r = 0.69, 95% CI 0.51 to 0.81, p < 0.0001.

**Figure 3:**
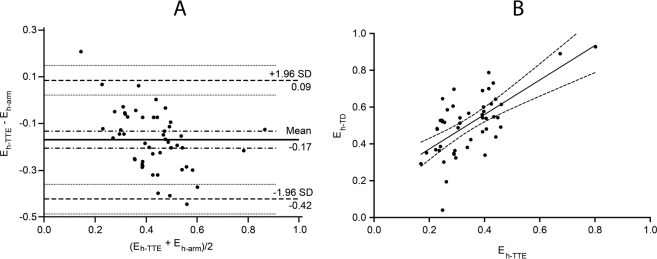
Graphic presentation of agreement and correlation between global heart efficiency estimated by the upper limb stop flow technique (E_h-arm_) and calculated using thermodilution measurements of cardiac output (E_h-TTE_). Panel A: The Bland Altman plot demonstrated a bias of −0.17 ± 0.12 (solid line) with the lower and upper limits of agreement at −0.42 and 0.09 (dashed lines). Panel B: Linear regression scatterplot graph. The correlation was r = 0.64, 95% CI 0.44 to 0.78, p < 0.0001.

The global heart efficiency calculated using echocardiography measurements of cardiac output was 0.35 ± 0.12. The Bland Altman analysis of global heart efficiency estimated by the upper limb stop-flow technique and by using transthoracic echocardiography measurements of cardiac output demonstrated a mean difference of −0.17 ± 0.12 with the lower and upper limits of agreement between −0.42 and 0.09. The correlation was r = 0.64, 95%CI 0.44 to 0.78, p < 0.0001. (Fig. 3).

**Figure 4:**
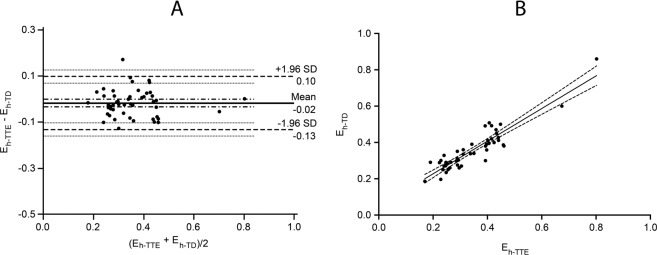
Agreement and correlation between global heart efficiency calculated based on cardiac output measurements using thermodilution (E_h-TD_) and global heart efficiency calculated based on cardiac output measurements using echocardiography (E_h-TTE_). Panel A: Bland-Altman plot demonstrated a bias of 0.02 ± 0.06 (solid line) with the lower and upper limits at −0.1 and 0.13 (dashed lines). Panel B: Linear regression scatterplot graph. The correlation was r = 0.87, 95% CI 0.78 to 0.93, p < 0.0001.

Bland-Altman analysis of global heart efficiency calculated based on cardiac output measurements using thermodilution methods and echocardiography demonstrated a mean difference of 0.02 ± 0.06 with the lower and upper limits between −0.1 and 0.13. The correlation was r = 0.87, 95% CI 0.78 to 0.93, p < 0.0001. (Fig. 4).

Agreements and correlations between calculations of global heart efficiency by three different techniques are presented in Table E5 in the Online Data Supplement.

Univariate analyses between the global heart efficiency variables, using estimates of mean systemic filling pressure from the stop-flow technique or the calculated analogue pressure based on thermodilution or echocardiographic cardiac output, and echocardiographic variables used to assess the cardiac systolic function are reported in Table 4. Left ventricular ejection fraction, left ventricular global longitudinal strain and tricuspid annular plane systolic excursion were the only investigated variables found to have moderate correlations.

**Table 4:** Univariate analysis of global heart efficiency and echocardiographic variables used for assessments of cardiac systolic function.

VARIABLE	E_h_ estimated by the upper limb stop-flow technique	E_h_ calculated using thermodilution measurements of CO	E_h_ calculated using echocardiographic measurement of CO
LV ejection fraction	r = 0.16 (p = 0.29)	**r** = **0.31** **(p** = **0.03)**	**0.32** **(p** = **0.03)**
LV dP/dt	rho = 0.06 (p = 0.68)	rho = 0.13 (p = 0.62)	rho = 0.10 (p = 0.71)
TAPSE	rho = 0.16 (p = 0.29)	**rho** = **0.37** **(p** = **0.01)**	**rho** = **0.36** **(p** = **0.01)**
RV strain	r = −0.16 (p = 0.41)	r = −0.27 (p = 0.15)	r = −0.31 (p = 0.09)
LV GLS	r = −0.05 (p = 0.77)	**r** = **−0.36** **(p** = **0.04)**	r = −0.26 (p = 0.15)

Correlations are described by Pearson (r) and Spearman (rho) with the p-values within brackets. Statistically significant results are depicted in bold.

Definition of abbreviations: Eh = global heart efficiency; LV = left ventricle; dP/dt = left ventricular maximal rate of systolic pressure rise; TAPSE = tricuspid annular plane systolic excursion; RV strain = right ventricular free wall longitudinal systolic strain; LV GLS = left ventricular global longitudinal strain.

The multivariate analysis of selected univariates did not demonstrate significant correlation between global heart efficiency and echocardiographic variables (see Table E6 in the Online Data Supplement).

## Discussion

This observational study did not demonstrate a ‘robust’ relationship between estimates of mean systemic filling pressure and static echocardiographic variables used in clinical practice to estimate volume status or between estimates of global heart efficiency and echocardiographic measurement of cardiac systolic function. These relationships were characterised by high indices of bias and imprecision between volume status estimates and moderate to weak correlations between cardiac performance indices.

The term ‘volume state’ refers to the static global cardiovascular filling, while volume responsiveness refers to the ability to increase cardiac output in response to the dynamic increase in filling. Mean systemic filling pressure is a physiological parameter representing volume status. This study was restricted to static echocardiographic measurements in relation to the volume state, although respiratory variation of inferior vena cava diameter which better reflects volume responsiveness is often clinically used to report volume state and was included in the analysis^15^.

Changes in the stressed intravascular volume will predominantly affect venous capacitance vessels with a smaller change in cardiac dimensions due to differences in compliance. While the inferior cava vein diameter may be influenced by the distending volume, the complex and dynamic interactions with right atrial pressure, determined by cardiac performance, intrathoracic and intraabdominal pressures, are significant^16,17^. These factors may explain the weak correlations between estimates of mean systemic filling pressure and echocardiographic variables.

Similar to a previous study^18^, our results demonstrated significant bias and imprecision for the upper limb stop-flow technique and analogue mean systemic pressure results, suggesting that these measurements are not interchangeable. The mean filling pressure measured in the arm compartment demonstrated the lowest correlation with any echocardiographic measurements of the volume state, its pathophysiological meaning remains uncertain. We therefore do not recommend the routine clinical use of static upper limb stop-flow pressure measurements.

Global heart efficiency is an estimate of the efficiency of the entire heart, while echocardiographic parameters quantify the performance of separate cardiac components at different stages of cardiac cycle. Global heart efficiency derived from analogue mean systemic filling pressure moderately correlated with left ventricular longitudinal strain, left ventricular ejection fraction and tricuspid annular plane systolic excursion. Tricuspid annular plane systolic excursion demonstrated the best correlation with global heart efficiency that may be consistent with the role of the right ventricle to maintain the gradient between mean systemic filling pressure and right atrial pressure. As our study did not examine stroke volume or cardiac output in relation to global heart efficiency due to the mathematical coupling in the calculations, these observations remain speculative.

The strengths of our study include a pre-specified protocol and statistical analysis plan. The study was conducted over a short inception period with high levels of data integrity. The enrolment criteria included a well-defined and clinically applicable intensive care patient population that allowed adequate statistical power to address the primary objective that is applicable for an explorative analysis. Confounding bias was mitigated by standardising investigative techniques and operator-dependent errors using echocardiography experts for image acquisition and analysis.

The limitations of this study include its observational nature and the use of indirect estimates of mean systemic filling pressure, instead of directly measured values. At the time of study, the patients were haemodynamically stabilized, with more than 70% receiving vasopressors, reflected by high mean values of the mean systemic filling pressure estimates. Most patients had echocardiographic signs of systolic and diastolic dysfunction, which limits external validity for patients without this pathology. For the purpose of the study, mean systemic filling pressure and heart efficiency were separately compared to echocardiographic parameters, but in clinical context they should arguably be viewed in conjunction, since variable contractility states can be associated with the entire spectrum of volume state. Measurements of strain are technically difficult in critically ill, which could contribute to the lack of identified correlation. We restricted the analysis to static variables that may inform the design of future studies of dynamic variables. The statistical power is limited by the sample size. We deliberately use conservative exploratory analyses to the interpretation of multivariate analyses to mitigate for Type 1 error.

To our knowledge there are no previous studies investigating relationships between mean systemic filling pressure and derived global heart efficiency, and echocardiographic variables that are widely used in clinical practice to assess volume status and cardiac systolic function.

Most previous research related to analogue mean systemic filling pressure was based on invasive or semi-invasive cardiac output measurements in cardiac surgery patients^18–21^. Our study demonstrates good potential to expand the use of analogue mean systemic filling pressure and derived global heart efficiency based on non-invasive echocardiographic measures of cardiac output to a wider cohort of intensive care patients provided that adequate echocardiographic views can be obtained in addition to mean arterial pressure and invasive central venous pressure. Moderate correlations between invasive and echocardiographic measurements of cardiac output in our investigation are aligned with the previous reports that these techniques are not interchangeable in intensive care patients^22^.

Our study provides caution to clinicians using and relying on static echocardiographic parameters to estimate volume status of their patients, particularly in dynamic situations and under conditions of limited echocardiographic imaging.

## Conclusions

Static echocardiographic variables did not reliably reflect the volume state as defined by estimates of mean systemic filling pressure. There was no statistical or clinically robust relationship between static echocardiographic variables of cardiac systolic function and global heart efficiency. Echocardiography remains valuable in estimating volume state by the ability to measure cardiac output for the calculation of analogue mean systemic filling pressure.

## Methods

We conducted a prospective, multi-centre observational study, the Comparative Haemodynamic Assessments using InvaSive and Echocardiographic techniques (CHAISE) study in adult patients treated in two university affiliated, multidisciplinary Intensive Care Units (ICUs) in Sydney, Australia. The study was approved (HREC/14/SVH/63) by the St Vincent’s Hospital (Sydney) Human Research and Ethics Committee and informed consent was obtained from all participants or their legal representatives. The study protocol and statistical analysis plan were finalised before data collection was initiated. All methods were performed in accordance with the relevant guidelines and regulations.

### Patients

Mechanically ventilated, sedated, adult patients admitted to ICU who required invasive monitoring of arterial pressure, central venous pressure and cardiac output as part of routine care were eligible for the study. Eligible patients were required to be haemodynamically stabilized for at least one hour, defined as being in sinus rhythm, no changes to vasoactive or inotropic therapy or requirement for fluid resuscitation during the observational study period and the attainment of adequate transthoracic echocardiographic views by an independent clinician. Patients were excluded if there was a contraindication or inability to apply an occlusive arm cuff, such as severe skin condition, humeral fracture, severe bleeding diathesis, lymphangioedema or high pain sensitivity.

### Measurements

All patients had a radial or brachial arterial catheter, central venous catheter and a thermodilution cardiac output catheter system *in situ* as determined by clinical indication. The pressure transducers were zeroed immediately before the study at the level of the fifth intercostal space in the anterior axillary line with patient positioned supine. Mechanical ventilation was conducted in synchronized IPPV mode with PEEP of 5–10 cm H_2_O. Haemodynamic parameters were recorded at the end-expiration of the ventilatory cycle. Cardiac output was measured using either pulmonary artery thermodilution (831VF75P, Edwards Lifesciences Pty Ltd, Sydney, Australia) with triplicate cold bolus injections within a ± 10% range or pulse contour analysis (PiCCO, Maquet, Rastatt, Germany) calibrated by triplicate transpulmonary thermodilution bolus injections within a ± 10% range. Transthoracic echocardiographic measurements of cardiac output were made simultaneously using standard techniques described below. The upper extremity rapid stop-flow technique to measure mean systemic filling pressure has been reported previously^8,18^. In brief, a rapid inflation narrow cuff was applied to the upper arm above the radial or brachial arterial catheter. The inflation pressure was set at 70 mmHg above the contemporarily measured invasive systolic pressure. An automated tourniquet system (A.T.S. 750, Zimmer Pty Ltd, Ashford, Australia) was used for rapid inflation with arterial flow occlusion occurring within one cardiac cycle. An equilibration time of 30 seconds was allowed before recording the pressure after which the cuff was deflated for 3 minutes. Three repeat measurements were performed, and a maximal 10% variation was accepted.

The analogue mean systemic filling pressure (P_msa_) was calculated according to the equation^9^:

$${{\rm{P}}}_{{\rm{msa}}}=(0.96\,\times \,{\rm{CVP}})+(0.04\,\times \,{\rm{MAP}})+({\rm{c}}\,\times \,{\rm{CO}})$$

where CVP is central venous pressure, MAP is mean arterial pressure, CO is cardiac output and c is a factor to adjust the influence of venous resistance according to the patient’s age, height and weight^12^ and defined as:

$$\begin{array}{rcl}{\rm{c}} & = & 0.038\times (94.17+0.193\,\times \,{\rm{age}})/(4.5\times [{0.9}^{({\rm{age}}-15)}]\,\times \,0.007184\\  &  & \times \,[{{\rm{height}}}^{0.725}]\,\times \,[{{\rm{weight}}}^{0.425}])\end{array}$$

Calculations of the analogue mean systemic filling pressure included results of simultaneous measurements of cardiac output using thermodilution and echocardiography. The pressure gradient for venous return was calculated by the difference between mean systemic filling pressure and central venous pressure.

Global heart efficiency (E_h_) was calculated according to the equation^9,12^:

$${{\rm{E}}}_{{\rm{h}}}=({{\rm{P}}}_{{\rm{ms}}}-{\rm{CVP}})/{{\rm{P}}}_{{\rm{ms}}}$$

where P_ms_ is mean systemic filling pressure and CVP is central venous pressure; E_h_ is a dimensionless ratio between zero and one, where zero describes a “no flow” state and one describes an optimal heart performance, with a typically observed range between 0.3 and 0.5 in critically ill patients^19,23^.

A physician qualified in transthoracic echocardiography (Advanced Transthoracic Echocardiography training, Level 2a or higher)^24^ performed all echocardiographic measurements (SC2000, Siemens Healthcare GmbH, Erlangen, Germany, or Sparq, Philips Ultrasound, Bothell, WA, USA). Images were acquired from parasternal, apical, subcostal transthoracic windows as per standard recommendations^16,25,26^. Cardiac output was calculated based on heart rate and measurements of the left ventricular outflow tract diameter and velocity time integral obtained with pulse wave Doppler. Three sets of velocity time integrals were averaged for each measurement. Early diastolic mitral annular velocity (e’) in the ratio E/e’ was calculated as an average of e’ measurements obtained from the medial and from the lateral mitral annulus. Inferior vena cava distensibility index (IVCDI) was calculated using the following equation:

$$IVCDI=\frac{(IVCmax-IVCmin)}{IVCmin}\times 100 \% $$

where *IVCmax* was maximum inferior vena cava diameter (measured during mechanical inspiration), *IVCmin* – minimum inferior vena cava diameter (measured during mechanical expiration).

The estimates of mean systemic filling pressure reflecting the volume state were evaluated against left ventricular end-diastolic volume/area and end-systolic volume/area as well as left and right atrial volumes, inferior vena cava dimensions and the ratio between early mitral inflow velocity and mitral annular early diastolic velocity (listed in Table 3). The estimates of heart efficiency reflecting cardiac systolic function were evaluated against left ventricular ejection fraction, LV maximal rate of systolic pressure rise and left ventricular global longitudinal strain as well as right ventricular free wall strain and tricuspid annular plane systolic excursion (listed in Table 4). Strain analysis of the left ventricle (LV), right ventricle (RV) and right atrium (RA) were performed on adequate quality images “on cart” or off-line using a research workstation (Syngo, Siemens Healthcare GmbH, Erlangen, Germany). RV strain was derived from the RV free wall, excluding ventricular septal segments.

### Statistical analyses

A sample size of 47 patients was calculated to detect a pre-specified correlation coefficient of 0.4 between echocardiographic indices and mean systemic pressure and global heart efficiency parameters^27^, using 80% power (alpha = 0.05). The sample size was increased to 50 to account for attrition during the study. Normal distribution of variables was assessed using the D’Agostino-Pearson test and continuous variables expressed as the mean ± standard deviation (SD) or the median and interquartile range (IQR) as appropriate. Correlations were assessed by Pearson’s (r) and Spearman (rho) coefficients for normally and non-normally distributed variables respectively. Correlations were pre-defined as “moderate” or “weak” with correlation coefficients of less than 0.6 and 0.3 respectively, regardless of the associated p value. A limits of agreement analysis, as defined by Bland-Altman, was used to compare estimates of mean systemic filling pressure, using the arm occlusion method and the calculated analogue pressure using cardiac output measurements from thermodilution and echocardiography as separate analyses, where the mean difference represents bias and the degree of dispersion (upper and lower 95% confidence intervals [95% CI]) represents precision. Multivariate regression analyses were conducted to determine the correlation between mean systemic filling pressure and echocardiographic measures of volume state and between global heart efficiency measurements and echocardiographic measures of cardiac systolic function. Cardiac output and stroke volume were excluded from the regression model of cardiac systolic function since they are mathematically coupled to the calculation of global heart efficiency. Variables were included in the multiple regression models when p < 0.2 from the initial analyses. The multiple regression, including the degrees of freedom and residual, are reported using the F-statistic, p-value and multiple correlation coefficients. A two-sided p-value less than 0.05 was considered statistically significant. No corrections were made for multiple comparisons.

A ‘robust’ relationship between variables was pre-defined as one with statistically significant low levels of bias and imprecision and correlation coefficients >0.7 where applicable. Statistical analyses were performed using MedCalc v 12.3 (MedCalc Software bvba, Ostend, Belgium).

## Supplementary information

Supplementary information.
