# Supplementary material for: Comparison of echocardiographic and invasive measures of volaemia and cardiac performance in critically ill patients
Source: Sci Rep. 2020 Mar 17;10:4863. doi: 10.1038/s41598-020-61761-1 (PMC7078248; doi:10.1038/s41598-020-61761-1)

Comparison of echocardiographic and invasive measures of volaemia and cardiac performance in critically ill patients.

Konstantin Yastrebov, Anders Aneman, Luis Schulz, Thomas Hamp, Peter McCanny, Geoffrey Parkin, John Myburgh.

Online Data Supplement

Table E1. Echocardiographic variables for the 50 patients studied. Values are mean (standard deviation) or median [interquartile range].

VARIABLE	Value
LV end-diastolic volume index (mL/m^2)	53 [45-70]
Short-axis mid-papillary LV end-diastolic area (cm^2)	17 [12-22]
LV end-systolic volume index (mL/m^2)	30 [20-42]
Short-axis mid-papillary LV end-systolic area (cm^2)	8 [5-16]
LV ejection fraction (%)	45 (16)
LA volume (mL/m^2)	31 [22-38]
RA volume (mL/m^2)	28 [20-33]
IVC diameter (inspiration) (mm)	17 (6.4)
IVC diameter (expiration) (mm)	17 (6.7)
IVC distensibility (%)	4 [2-8]
Early mitral diastolic inflow velocity (cm/sec)	91 (24)
Late mitral diastolic inflow velocity (cm/sec)	65 [48-87]
Early diastolic medial mitral annular velocity (cm/sec)	6.3 (1.9)
Early diastolic lateral mitral annular velocity (cm/sec)	8.2 (2.6)
Early tricuspid diastolic inflow velocity (cm/sec)	50 [41-62]
Late tricuspid diastolic inflow velocity (cm/sec)	44 [36-52]
Early diastolic lateral tricuspid annular velocity (cm/sec)	6.4 [5.5-8.0]
Tricuspid annular plane systolic excursion (cm)	12 [10-16]
Global longitudinal LV strain (%)	-10 (3.8)
RV free wall systolic strain (%)	-12 (4.5)
RA strain (%)	18 [11-27]
Rate of rise in LV pressure (mm Hg/sec)	857 [660-1393]

Definition of abbreviations: LV = left ventricle; LA = left atrium; RA = right atrium; IVC = inferior vena cava; RV = right ventricle

Table E2: Estimation of mean systemic filling pressure and global heart efficiency by three different techniques.

	**Upper limb stop-flow technique measurements**	**Analogue estimates using Thermodilution technique to measure cardiac output**	**Analogue estimates using Echocardiography technique to measure cardiac output**
**P_{ms} mean ± SD (mmHg)**	26±5.2	19±3.9	18.5±3.7
**E_h mean ± SD**	0.51±0.17	0.36±0.12	0.35±0.12

Definition of abbreviations: P_{ms} = mean systemic filling pressure; E_h = global heart efficiency

Table E3: Agreement and correlation between estimates of mean systemic filling pressure by three different techniques.

	**Bias (mmHg) ± SD**	**Levels of agreement (mmHg)**	**Correlation (r)**	**95% CI**	***p*-value for r**
**Upper limb stop-flow technique *vs* Analogue estimates using Thermodilution technique to measure cardiac output**	-6.9±0.84	-18 to 4.6	0.19	-0.1 to 0.44	0.20
**Upper limb stop-flow technique *vs* Analogue estimates using Echocardiography technique to measure cardiac output**	-7.46±6.1	-19 to 4.5	0.11	-0.18 to 0.37	0.48
**Analogue estimates using Thermodilution technique to measure cardiac output *vs* Analogue estimates using Echocardiography technique to measure cardiac output**	0.52±1.7	-2.9 to 3.9	0.90	0.82 to 0.94	<0.001

Table E4. Multivariate analysis of mean systemic filling pressure and selected echocardiographic variables used for assessment of fluid status. Correlations are described by the F-statistic with regression and residual degrees of freedom in brackets, the p-value and the adjusted regression coefficient.

VARIABLE	P_{ms} estimated by the upper limb stop-flow technique	P_{ms} calculated using thermodilution measurements of CO	P_{ms} calculated using echocardiographic measurement of CO
**LV end-diastolic volume index (ml/m²)**<br>**LV end-systolic volume index (ml/m²)**<br>**RA volume index (ml/m²)**<br>**IVC diameter (inspiration) (mm)**<br>**IVC diameter (expiration) (mm)**<br>**IVC distensibility index (%)**<br>**E/e'**	F (7,37) = 0.94<br>p=0.47<br>r=0.33	F (7,35) = 1.16<br>p=0.35<br>r=0.43	F (7,37) = 0.38<br>p=0.57<br>r=0.38

Definition of abbreviations: P_{ms} = mean systemic filling pressure; CO = cardiac output; LV = left ventricle; RA = right atrium; IVC = inferior vena cava; E/e' = early mitral diastolic inflow velocity to early diastolic mitral annular motion velocity ratio.

Table E5: Agreement and correlation between estimates of global heart efficiency by three different techniques.

	Bias (mmHg) ± SD	Levels of agreement (mmHg)	Correlation (r)	95% CI	p-value for r
**Upper limb stop-flow technique Vs Analogue estimates using Thermodilution technique to measure cardiac output**	-0.15±0.12	-0.39 and 0.09	0.69	0.51 to 0.81	<0.0001
**Upper limb stop-flow technique Vs Analogue estimates using Echocardiography technique to measure cardiac output**	-0.17±0.12	-0.42 and 0.09	0.64	0.44 to 0.78	<0.0001
**Analogue estimates using Thermodilution technique to measure cardiac output vs Analogue estimates using Echocardiography technique to measure cardiac output**	0.02±0.06	0.1 and 0.13	0.87	0.78 to 0.93	<0.0001

Table E6. Multivariate analysis of global heart efficiency and selected echocardiographic variables used for assessment of cardiac systolic function. Correlations are described by the F-statistic with regression and residual degrees of freedom in brackets, the p-value and the adjusted regression coefficient

VARIABLE	E_h estimated by the upper limb stop-flow technique	E_h calculated using thermodilution measurements of CO	E_h calculated using echocardiographic measurement of CO
**LV ejection fraction**<br>**TAPSE**<br>**RV strain**<br>**LV GLS**	F (4,26) = 0.23<br>p=0.92<br>r=0.19	F (4,26) = 1.16<br>p=0.35<br>r=0.40	F (4,26) = 1.22<br>p=0.33<br>r=0.40

Definition of abbreviations: Eh = global heart efficiency; LV = left ventricle; TAPSE = tricuspid annular plane systolic excursion; RV strain = right ventricular free wall longitudinal systolic strain; LV GLS = left ventricular global longitudinal strain.

Figure E1. Study CONSORT diagram.

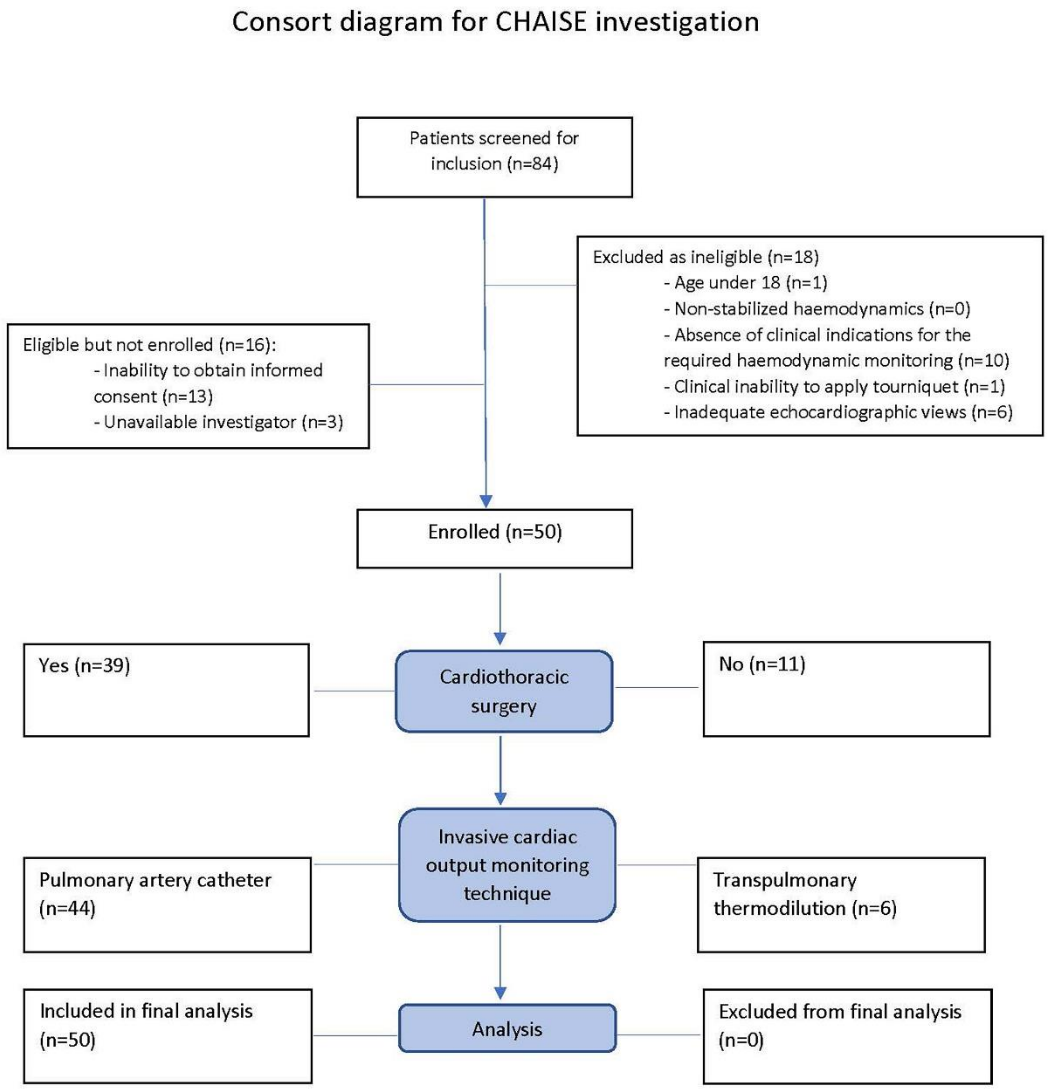

Supplement: Supplementary file 1 — Supplementary information. [file 41598_2020_61761_MOESM1_ESM.pdf]
